# Larvicidal and antimicrobial activities of silver nanoparticles synthesized using marine fluorescent pseudomonads

**DOI:** 10.1186/1471-2334-14-S3-P25

**Published:** 2014-05-27

**Authors:** Shanmugaiah Vellasamy, Harikrishnan Hariharan, TS Venkatesh, Mani Jayaprakashvel

**Affiliations:** 1 Department of Microbial Technology, School of Biological Sciences, Madurai Kamaraj University, Madurai-625021, India; 2 Department of Biotechnology, Anna University of Technology, Tiruchirappalli-620024, India; 3 Department of Biotechnology, AMET University, Kanathur, Chennai-603112, India

## Background

Research on nanoparticles opens up newer avenues for unraveling various biological challenges including the control of infectious diseases. The present investigation has been aimed to study larvicidal and antimicrobial activities of silver nanoparticles (AgNP’s) synthesized using marine fluorescent pseudomonads.

## Methods

In the present study, marine fluorescent pseudomonads were screened against various human pathogenic bacteria and plant fungal pathogens. An autolyzed cell free culture filtrate of selected isolate was used for the synthesis of silver nano particles and characterized using UV, FTIR, XRD and AFM analysis. The antimicrobial efficiency along with larvicidal activity of the synthesized nanoparticles was carried out by observing the lifecycle of mosquito for 96 hours along with appropriate controls.

## Results

Atomic microscopic observation showed that the particle size ranges from 10- 100nm. The peaks in UV spectrum clearly indicate that the particles are in the nanoregime. FTIR measurements explained the reduction of silver ions and stabilization of silver nanoparticles. At 96 hours, 60% mortality observed in test, while only 10% mortality observed in the control. AgNP’s showed significant activity against tested human pathogens and plant fungal pathogens.

## Conclusion

With the suppression of growth of filarial nematode in the larval stage, AgNP’s has the potential in eradicating this vector borne disease. It is clear that this isolate can be used to synthesize bioactive nanoparticles efficiently in an eco friendly and cost effective manner besides the fact that it can be used for the control of human and plant pathogenic microbes.

